# FDG PET/CT in Initial Staging of Adult Soft-Tissue Sarcoma

**DOI:** 10.1155/2012/960194

**Published:** 2012-12-02

**Authors:** David Roberge, Siavosh Vakilian, Yazan Z. Alabed, Robert E. Turcotte, Carolyn R. Freeman, Marc Hickeson

**Affiliations:** ^1^Department of Radiation Oncology, McGill University Health Centre, 1650 Cedar Avenue, Montreal, QC, Canada H3G 1A4; ^2^Department of Radiation Oncology, Notre-Dame Hospital, CHUM, 1560 Sherbrooke Street East, Montreal, QC, Canada H2L 4M1; ^3^Department of Nuclear Medicine, McGill University Health Centre, 1650 Cedar Avenue, Montreal, QC, Canada H3G 1A4; ^4^Department of Orthopaedic Surgery, McGill University Health Centre, 1650 Cedar Avenue, Montreal, QC, Canada H3G 1A4

## Abstract

Soft-tissue sarcomas spread predominantly to the lung and it is unclear how often FDG-PET scans will detect metastases not already obvious by chest CT scan or clinical examination. Adult limb and body wall soft-tissue sarcoma cases were identified retrospectively. Ewing's sarcoma, rhabdomyosarcoma, GIST, desmoid tumors, visceral tumors, bone tumors, and retroperitoneal sarcomas were excluded as were patients imaged for followup, response assessment, or recurrence. All patients had a diagnostic chest CT scan. 109 patients met these criteria, 87% of which had intermediate or high-grade tumors. The most common pathological diagnoses were leiomyosarcoma (17%), liposarcoma (17%), and undifferentiated or pleomorphic sarcoma (16%). 98% of previously unresected primary tumors were FDG avid. PET scans were negative for distant disease in 91/109 cases. The negative predictive value was 89%. Fourteen PET scans were positive. Of these, 6 patients were already known to have metastases, 3 were false positives, and 5 represented new findings of metastasis (positive predictive value 79%). In total, 5 patients were upstaged by FDG-PET (4.5%). Although PET scans may be of use in specific circumstances, routine use of FDG PET imaging as part of the initial staging of soft-tissue sarcomas was unlikely to alter management in our series.

## 1. Introduction

Soft-tissue sarcomas are a histologically heterogeneous group of malignant tumors. They are uncommon tumors, accounting for only 0.7% of adult malignancies and approximately 6.5% of childhood cancers. In 2011, 10,980 new soft-tissue sarcomas were expected to be diagnosed in the USA with 3,920 deaths expected from these tumors [1]. 

Soft-tissue sarcomas have a propensity for hematogenous metastases, the risk of which correlates with tumor size, grade, location, and histological subtype [2]. The most common site of metastasis, both at presentation and at recurrence, is the parenchyma of the lung. As pulmonary lesions account for approximately 75% of all metastases [3], the utility of adding abdomino-pelvic imaging has been debated [4]. There may be exceptions to this pattern of spread, such as in the case of myxoid or round cell liposarcomas that are known to have a greater propensity for retroperitoneal and bony metastases [5, 6].

PET/CT imaging has been investigated in soft-tissue sarcoma for biopsy guidance [7], response assessment [8], grading [9], followup [10], and prognostication [11]. Although the sensitivity of PET/CT for the identification of primary soft-tissue sarcoma tumors is well known [12, 13], different studies have reported wide-ranging sensitivities and specificities for the detection of metastatic disease [14–16]. The utility, beyond conventional staging, of FDG-PET/CT for the initial staging of soft-tissue sarcomas remains to be defined [17, 18]. In our institution, FDG-PET/CT imaging has been routinely performed in patients with large (AJCC T2) or high-grade sarcomas (FNLCC grade 2-3) at the time of diagnosis. These studies were performed as part of a larger, ethics review board approved, prospective study investigating the safety and utility of FDG in imaging of cancer patients.

## 2. Materials and Methods

### 2.1. Patients

A review of the database of the PET/CT unit of the Department of Nuclear Medicine of the McGill University Health Centre from May 2004 to November 2010 revealed 345 patients imaged with total body FDG-PET for a soft tissue or bone sarcoma. Patients with Ewing's sarcoma, rhabdomyosarcoma, gastrointestinal stromal tumors, desmoid tumors, bone tumors, visceral tumors and retroperitoneal sarcomas were excluded, as were patients imaged for followup, response assessment, or recurrence. This left 109 patients who had had a PET/CT study for the initial staging of their extremity or body wall soft-tissue sarcoma. The hospital charts of these patients were reviewed for pertinent clinical information.

### 2.2. Conventional Staging Studies

In addition to imaging (typically MRI) of the primary tumor site, every patient underwent a dedicated CT study of the chest. Patients underwent additional studies at the treating physician's discretion, typically to investigate symptoms or clinical findings suspicious for metastatic disease. The clinical reports used for patient care were used as the basis for considering a study as positive, negative, or indeterminate. An indeterminate study was typically a chest CT on which millimetric parenchymal and/or pleural nodules were seen. 

The CT scans of the chest, corroborated by histological diagnoses or subsequent clinical course, were considered the gold standard for determination of the presence or absence of metastasis.

### 2.3. FDG-PET/CT Scanning

Following written informed consent, FDG-PET studies were acquired on the hybrid PET/CT scanner (Discovery ST, General Electric Medical Systems, Waukesha, WI, USA), which combines a dedicated, full-ring PET scanner with a 16-slice spiral CT scanner. Between 370 and 500 MBq (10 and 13.5 mCi) of FDG was injected intravenously. Sixty minutes following FDG injection, CT and PET images were consecutively acquired from the base of the skull to the upper thighs, with additional images acquired according to the sarcoma location. In the PET portion of the study, a 2D acquisition was performed and images were acquired using 4-5 min per bed position (depending on the body weight) with 5 to 6 bed positions (depending on the patient's height). PET attenuation corrected, PET nonattenuation corrected, CT, and fused images were reconstructed in the transaxial, coronal, and sagittal planes with an ordered subset expectation maximization (OSEM) iterative algorithm. 

In those patients who still had their primary tumor in place, the SUVmax values were measured using a rounded region of interest tool and searching systematically slice by slice for the most intense voxel within a given lesion. The primary tumor was considered PET positive if it had a SUVmax of 2.5 or greater. The clinical reports used for patient care were used as the basis of considering the study positive, negative, or indeterminate for the presence of metastatic disease. An indeterminate study was a study on which an FDG avid lesion was seen which was considered atypical for a metastasis or a nonavid abnormality was seen on the CT component of the study.

## 3. Results

From May 2004 to November 2010, 109 patients underwent total body FDG PET/CT imaging as part of the initial staging of a soft-tissue sarcoma. The patient and tumor characteristics are presented in Table 1. Nineteen percent of patients had had their primary tumor removed by excisional biopsy or unplanned excision prior to staging. Of the previously unresected primary tumors, 98% were FDG avid (median SUVmax 7.7, range 1.7–35.8). Eighty-seven percent of tumors were intermediate or high grade (FNCLCC grade 2-3). The primary tumor was stage T2b in 64% of cases. The most common primary site was the lower extremity (66%). The most common pathological diagnoses were leiomyosarcoma (17%), liposarcoma (17%), undifferentiated or pleomorphic (16%), fibrosarcoma (16%), synovial sarcoma (12%), malignant peripheral nerve sheath tumor (10%), and epitheliod sarcoma (6%). 

**Figure 1:**
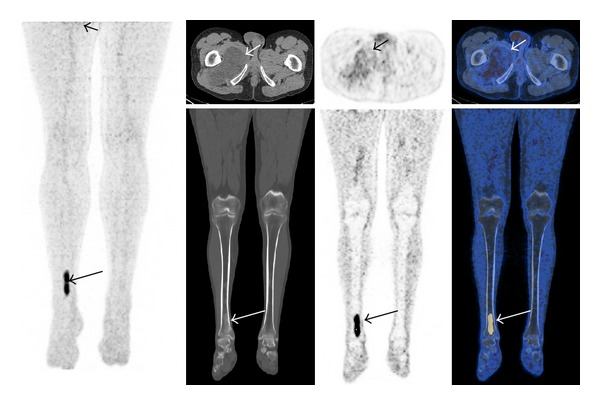
False positive 48 year old with a large myxoid liposarcoma of the right thigh (short arrow) with low metabolic activity (SUV 2.6) required biopsy to demonstrate that an avid (SUV 7.4) lesion of the right distal tibia (long arrow) was fibrous dysplasia.

**Figure 2:**
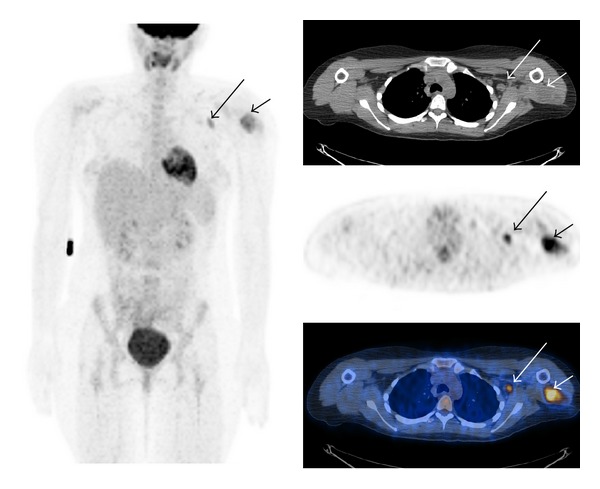
False Positive a 29 year old with an angiomatoid malignant histiocytoma of the left deltoid (short arrow) with an SUV of 6.5. The FDG-PET scan demonstrated a hypermetabolic left axillary lymph node (long arrow) with an SUV of 4.4. The latter was biopsied and was a reactive lymph node on final pathology.

PET scans were negative for distant disease in 91/109 cases. Ten of these 91 cases had metastatic disease on chest CT (false negative). The negative predictive value of the PET was 89% and the specificity was 96%. Fourteen patients had positive PET scans. Of these, 6 were in patients already known to have metastases, 3 were false positives, and 5 were new findings of metastatic disease. All false positive studies resulted in additional interventions: in one case an open biopsy of tibial fibrous dysplasia (Figure 1), in another, axillary dissection of reactive lymph nodes (Figure 2), and the other, needle biopsy of reactive right iliac nodes. Four patients had indeterminate PET scans. With a followup of 13–27 months, none of those four developed metastatic disease. Two incidental benign parotid tumors were found. PET scanning also discovered an incidental non-small-cell lung cancer that had not clearly been identified on the chest CT, which had reported an indeterminate 8 mm parenchymal nodule and nonspecific mediastinal adenopathy.

**Figure 3:**
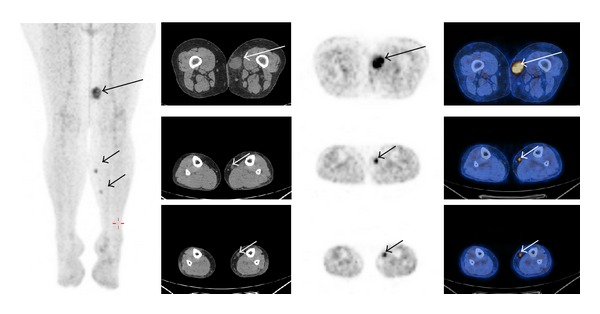
False negative a 35-year-old patient with a large round cell liposarcoma of the right ankle (top, small arrow) required nephrectomy for a large infrarenal mass (bottom, long arrow) which in retrospect was a non-FDG-avid lesion on the staging FDG-PET scan (middle, long arrow).

At the end of staging, 19% of patients were considered to have metastatic disease. Of these 21 patients, 16 had pulmonary metastases. In retrospect, one patient with a large round cell liposarcoma of the ankle (Figure 3) had a 1.4 cm non-avid suprarenal lymph node on the CT portion of the PET/CT. The lesion, not mentioned on the PET report, was substantially larger on a follow-up study. On resection, it was confirmed to be a metastatic lesion and the original PET study is counted in this series as a false negative.

**Figure 4:**
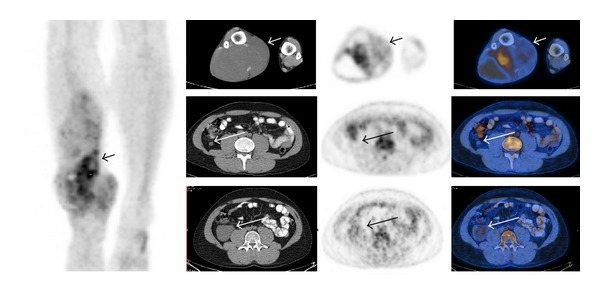
True positive in this 58-year-old patient with malignant peripheral nerve sheath tumor of the right thigh (long arrow). The FDG-PET scan demonstrated two additional hypermetabolic lesions in the left calf (short arrow). These were subsequently resected and proven to be synchronous peripheral nerve sheath tumors. Four years later, the patient is in remission and is doing well.

**Figure 5:**
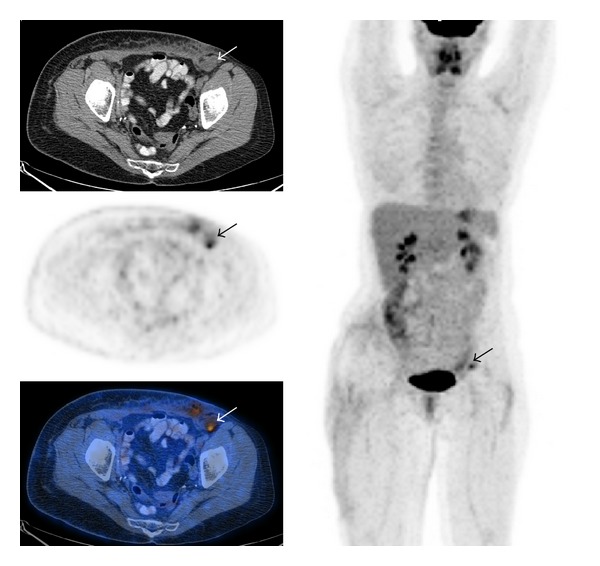
True positive that changed management; in 45-yea-old patient with a recently resected left lower abdominal wall angiosarcoma, PET/CT demonstrated uptake in the left inguinal region (arrow). This was subsequently biopsied and proven to be metastatic angiosarcoma.

In total, 5 patients were upstaged by the PET imaging (4.5%): of these, 3 had extrathoracic metastases only, one had lung metastases not identified by chest CT, and one patient had both extrathoracic and lung metastases. The finding of metastatic disease resulted in inguinal node biopsy, followed by inguinal node dissection and radiotherapy in one patient, resection of soft tissue metastases in the lower limb in another, and addition of palliative chemotherapy for the 3 other patients. All 5 patients underwent surgical resection of the primary tumor, which for one patient required an amputation, and in another required metastasectomy of soft tissue and lung metastases. All 4 patients were alive at last followup. Two patients have no evidence of recurrent disease, 2 patients have progressive metastatic disease to the lungs and are undergoing palliative chemotherapy, and one patient has stable residual metastatic disease. Of note, one particularly interesting patient with a malignant peripheral nerve sheath tumor of the thigh, mentioned above, was found on PET/CT to have two soft-tissue metastases to the lower extremity, resulting in surgical excision (Figure 4). The PET, as well as the CT chest, also demonstrated a suspicious-looking pulmonary nodule, which eventually grew and proved to be metastatic. She underwent surgical excision of the lung lesion as well and received systemic chemotherapy; she currently has no evidence of recurrent disease. A true positive PET of unsuspected extrathoracic disease is illustrated in Figure 5. 

Finally, PET did not alter management of patients already known to have M1 disease. The sensitivity of the PET/CT for the detection of metastatic disease was 52% and the positive predictive value 79% (Table 2).

## 4. Discussion

There are few other reports in which metabolic imaging was used in the initial staging of soft-tissue tumors. Moreover, these series tend to include heterogenous diagnoses as well as bone tumors.

In a retrospective study, Tateishi et al. [19] reviewed the images of 117 patients having undergone staging for a suspected bone or soft-tissue tumor. In addition to conventional imaging which included technetium-99 m-HMDP bone scintigraphy, chest radiography, and total body CT, an FDG-PET scan was performed in each case. The metabolic imaging found distant metastases in an additional 14% beyond conventional imaging. The anatomical site of these metastases was not given. Of note, too, 41% of the patients had metastases and the series included osseous tumors as well as soft-tissue osteosarcomas and Ewing's sarcomas which were excluded from our study.

In another retrospective study, Iagaru et al. [20] reported on 44 patients with osseous and soft-tissue sarcomas imaged with combination PET/CT. The CT and metabolic portions of the scan were reviewed separately. PET was found to be less sensitive than CT for the detection of metastases 78.6% versus 82.3% but more specific 92.8% versus 76%. In addition to bone tumors and Ewing's sarcomas, this series included rhabdomyosarcomas. From the manuscript, it was not possible to tell if any patient would have been upstaged by the addition of metabolic imaging.

In a small series of 16 patients imaged for initial staging of a bone or soft-tissue sarcoma, Piperkova et al. reported that no additional metastatic lesions were detected by metabolic imaging versus CT imaging [21].

Our results are not surprising in view of the prevalence of distant metastases in our patient population and the expected patterns of spread of soft-tissue sarcoma. If the pretest probability of having metastatic disease is 20% and 75% of metastatic lesions will be intrathoracic (where CT is more sensitive [22]), a yield of less than 5% is not unexpected, especially if one accounts for symptomatic lesions, lesions visible on conventional imaging and false negatives. Even when excluding all low-grade tumors, all small tumors (T1), and all superficial tumors in our series, management was altered in only 3.3% of cases. Histologies were different for all the 5 cases where management was altered; however, none were low grade. Although it would seem appropriate to increase the yield of PET staging by limiting its use to patients with a higher expected proportion of extrathoracic metastases, this is not necessarily a straightforward proposition. For example, although myxoid liposarcoma is known to have a higher propensity for bony metastases, MRI may be more useful for screening [5]. In the selected clinical scenarios, the PET may be justified despite the low yield because of a large impact on clinical care. 

In our series, three false positive and three indeterminate PET findings resulted in additional investigations and surgical procedures. Beyond the costs incurred, such findings can lead to delayed management of the primary tumor and additional patient morbidity.

To our knowledge, this is the largest and most homogeneous series of patients who have undergone FDG-PET imaging as initial staging for adult limb and body wall soft-tissue sarcoma. When pediatric-type tumors, bone tumors, and visceral primaries are excluded, the incremental information provided over standard chest imaging was found to be limited. In contrast to non-small-cell lung cancer, where the argument for PET staging can be made on a purely economic basis, no costly surgery was averted in our series. Although the benefit is difficult to quantify, those patients managed more aggressively for their PET-detected metastatic disease appear to have benefited from early aggressive management.

## 5. Conclusion

Although we have found 98% of primary adult soft-tissue sarcomas to be FDG-avid, the use of routine FDG PET imaging for detection of metastatic disease as part of initial staging for soft-tissue sarcoma altered management in less than 5% of our patients.

## Figures and Tables

**Table 1:** Patient and tumor characteristics.

Patient age (median)	55 (range 12–90)
Patient gender	
Female	52%
Male	48%
Primary tumor stage	
T1a	21%
T1b	8%
T2a	7%
T2b	64%
Tumor grade	
TNM low (FNLCC grade 1)	9%
TNM high (FNLCC grade 2-3)	87%
Unknown/ungradable	4%
Tumor location	
Lower extremity	66%
Upper extremity	23%
Body wall	11%
Tumor histology	
Leiomyosarcoma	17%
Liposarcoma	17%
Fibrosarcoma	16%
Undifferentiated	16%
Synovial sarcoma	12%
MPNST	10%
Epitheliod	6%
Other	7%

**Table 2:** PET results.

True negative	81 (74%)
False negative	10 (9%)
True positive	
Known	6 (5.5%)
New	5 (4.5%)
False positive	3 (3%)
Indeterminate	4 (4%)

## References

[1] Siegel R, Ward E, Brawley O (2011). Cancer statistics, 2011: the impact of eliminating socioeconomic and racial disparities on premature cancer deaths. *CA: A Cancer Journal for Clinicians*.

[2] Mariani L, Miceli R, Kattan MW (2005). Validation and adaptation of a nomogram for predicting the survival of patients with extremity soft tissue sarcoma using a three-grade system. *Cancer*.

[3] Potter DA, Glenn J, Kinsella T (1985). Patterns of recurrence in patients with high-grade soft-tissue sarcomas. *Journal of Clinical Oncology*.

[4] King DM, Hackbarth DA, Kilian CM, Carrera GF (2009). Soft-tissue sarcoma metastases identified on abdomen and pelvis CT imaging. *Clinical Orthopaedics and Related Research*.

[5] Conill C, Setoain X, Colomo L (2008). Diagnostic efficacy of bone scintigraphy, magnetic resonance imaging, and positron emission tomography in bone metastases of myxoid liposarcoma. *Journal of Magnetic Resonance Imaging*.

[6] Moreau LC, Turcotte R, Ferguson P (2012). Myxoid\round cell liposarcoma (MRCLS) revisited: an analysis of 418 primarily managed cases. *Annals of Surgical Oncology*.

[7] Hicks RJ, Toner GC, Choong PF (2005). Clinical applications of molecular imaging in sarcoma evaluation. *Cancer Imaging*.

[8] Benz MR, Czernin J, Allen-Auerbach MS (2009). FDG-PET/CT imaging predicts histopathologic treatment responses after the initial cycle of neoadjuvant chemotherapy in high-grade soft-tissue sarcomas. *Clinical Cancer Research*.

[9] Folpe AL, Lyles RH, Sprouse JT, Conrad EU, Eary JF (2000). (F-18) fluorodeoxyglucose positron emission tomography as a predictor of pathologic grade and other prognostic variables in bone and soft tissue sarcoma. *Clinical Cancer Research*.

[10] Bastiaannet E, Groen B, Jager PL (2004). The value of FDG-PET in the detection, grading and response to therapy of soft tissue and bone sarcomas; a systematic review and meta-analysis. *Cancer Treatment Reviews*.

[11] Eary JF, O’Sullivan F, Powitan Y (2002). Sarcoma tumor FDG uptake measured by PET and patient outcome: a retrospective analysis. *European Journal of Nuclear Medicine*.

[12] Roberge D, Hickeson M, Charest M, Turcotte RE (2010). Initial McGill experience with fluorodeoxyglucose PET/CT staging of soft-tissue sarcoma. *Current Oncology*.

[13] Charest M, Hickeson M, Lisbona R, Novales-Diaz JA, Derbekyan V, Turcotte RE (2009). FDG PET/CT imaging in primary osseous and soft tissue sarcomas: a retrospective review of 212 cases. *European Journal of Nuclear Medicine and Molecular Imaging*.

[14] Lucas JD, O’Doherty MJ, Wong JCH (1998). Evaluation of fluorodeoxyglucose positron emission tomography in the management of soft-tissue sarcomas. *Journal of Bone and Joint Surgery B*.

[15] Schwarzbach MHM, Dimitrakopoulou-Strauss A, Willeke F (2000). Clinical value of [18-F] fluorodeoxyglucose positron emission tomography imaging in soft tissue sarcomas. *Annals of Surgery*.

[16] Johnson GR, Zhuang H, Khan J, Chiang SB, Alavi A (2003). Roles of positron emission tomography with fluorine-18-deoxyglucose in the detection of local recurrent and distant metastatic sarcoma. *Clinical Nuclear Medicine*.

[17] Kumar R, Chauhan A, Vellimana AK, Chawla M (2006). Role of PET/PET-CT in the management of sarcomas. *Expert Review of Anticancer Therapy*.

[18] Toner GC, Hicks RJ (2008). PET for sarcomas other than gastrointestinal stromal tumors. *Oncologist*.

[19] Tateishi U, Yamaguchi U, Seki K, Terauchi T, Arai Y, Kim EE (2007). Bone and soft-tissue sarcoma: preoperative staging with fluorine 18 fluorodeoxyglucose PET/CT and conventional imaging. *Radiology*.

[20] Iagaru A, Quon A, McDougall IR, Gambhir SS (2006). F-18 FDG PET/CT evaluation of osseous and soft tissue sarcomas. *Clinical Nuclear Medicine*.

[21] Piperkova E, Mikhaeil M, Mousavi A (2009). Impact of PET and CT in PET/CT studies for staging and evaluating treatment response in bone and soft tissue sarcomas. *Clinical Nuclear Medicine*.

[22] De Wever W, Meylaerts L, Ceuninck L, Stroobants S, Verschakelen JA (2007). Additional value of integrated PET-CT in the detection and characterization of lung metastases: correlation with CT alone and PET alone. *European Radiology*.

